# Cost of inappropriate antimicrobial use for upper respiratory infection in Japan

**DOI:** 10.1186/s12913-020-5021-1

**Published:** 2020-02-28

**Authors:** Shinya Tsuzuki, Yuki Kimura, Masahiro Ishikane, Yoshiki Kusama, Norio Ohmagari

**Affiliations:** 1 AMR Clinical Reference Center, National Center for Global Health and Medicine, 1-21-1 Toyama, Shinjuku-ku, Tokyo, 162-8655 Japan; 2 Faculty of Medicine and Health Sciences, University of Antwerp, Antwerp, Belgium; 3 Disease Control and Prevention Center, National Center for Global Health and Medicine, Tokyo, Japan

**Keywords:** URI, Antimicrobial consumption, Cost analysis

## Abstract

**Background:**

Antibiotics are often prescribed inappropriately to patients with upper respiratory infection (URI) in ambulatory care settings; however, the economic burden of such prescription has not been quantitatively assessed. Here, we aimed to evaluate the additional cost of antimicrobial prescription for URI at the population level in Japan.

**Methods:**

We conducted a retrospective observational survey using longitudinal claims data between 2013 and 2016 obtained from JMDC Claims Database, which contains data from 5·1 million corporate employees and family members under the age of 65 years. Appropriateness of antibiotic prescription was assessed by a panel of six infectious disease physicians according to ICD-10 code in JMDC Claims Database. Total additional cost of antibiotic prescription for URI at the national level was estimated by weighting of age-structured population data.

**Results:**

The annual additional cost of inappropriate antibiotic prescription for URI was estimated at 423·6 (95% CI: 416·8–430·5) million USD in 2013, 340·9 (95% CI: 335·7–346·2) million USD in 2014, 349·9 (95% CI: 344·5–355·3) million USD in 2015, and 297·1 (95% CI: 292·4–301·9) million USD in 2016. Three classes of broad-spectrum oral antibiotics (third-generation cephalosporins, macrolides, and fluoroquinolones) accounted for > 90% of the total additional cost.

**Conclusions:**

Although a decreasing trend was observed, annual additional costs of inappropriate antibiotic prescriptions for URI could be a substantial economic burden in Japan. Appropriately prescribing broad-spectrum oral antibiotics might be an important issue to reduce unnecessary medical costs in Japanese ambulatory care.

## Background

Antibiotic prescription promotes antimicrobial resistance (AMR), which is currently one of the greatest threats to global health [1]. Antibiotic consumption is one of the key drivers of AMR [2, 3]; thus, reasonable prescription should be an important strategy of AMR countermeasures. Nevertheless, antibiotics are often prescribed inappropriately to patients with upper respiratory infection (URI) in ambulatory care settings [4, 5].

Following the publication of the Global Action Plan on Antimicrobial Resistance in 2015 [1], the Japanese government established the National Action Plan on Antimicrobial Resistance in 2016 [6]. According to this plan, reduction of oral antibiotics is specified as one of the main outcome indices for the evaluation of AMR countermeasures in Japan. A previous study estimated that physicians have prescribed antibiotics for about 60% of URI cases [7]. However, the trend of prescription behaviour of inappropriate antibiotic use for URI in ambulatory care remains unclear.

Additionally, antibiotic prescription for URI might impose a significant burden on our society because although URI is not a severe disease at the individual level, the total cost of antimicrobial use for URI does not seem to be negligible. Furthermore, AMR caused by such antimicrobial use will result in additional AMR infections and these cases will impose significant additional costs.

Although there are some previous studies about the additional cost of antimicrobial prescription for URI in ambulatory care settings, these studies focused on more extensive kind of drugs or diseases or specific populations [8–10]. Therefore, the main objective of the present study is to clarify the amount of additional cost due to inappropriate antibiotic prescription for URI and its trend in recent years.

## Methods

### Data source

We conducted a retrospective observational survey using longitudinal claims data collected between 2013 and 2016 obtained from JMDC Claims Database, which contains anonymous, de-identified claims data of 5·1 million corporate employees who are covered by the employees’ health insurance plan and their family members under the age of 65 years.

### Assessment of appropriate antibiotic prescription

We identified the number of claims that included inappropriate antibiotic prescription for URI patients. URI patients were defined by claims with the International Classification of Diseases, 10th Revision (ICD-10) diagnosis code of J00–06 and/or J20–22 without other diagnosis considered as appropriate for antimicrobial treatment.

To judge appropriate antibiotic prescribing behaviour for other ICD-10 codes, a panel of six infectious disease doctors at the Japanese Disease Control and Prevention Center, National Center for Global Health and Medicine, assessed the appropriateness of antibiotic prescription for each ICD-10 code. Each physician first independently evaluated whether treatment with antibiotics was optimal. If two or more assessors did not reach an agreement for any diagnosis code, then all six experts reviewed the case at a round table until a consensus was reached. The validity of this approach has been shown in previous studies [11, 12]. Details of the discussion process to judge appropriateness of antibiotic prescription are available in Supplementary file 1. As a result of the discussion, 75 ICD-10 codes are classified as “inappropriate” for antibiotic use. Details of ICD-10 codes classification are available in Supplementary file 2.

### Cost analysis

The total additional cost of inappropriate antibiotic prescription at the national population level was estimated by the JMDC data, which include the duration and cost of each drug prescribed in each claim. The cost of inappropriate antibiotic prescription in each age group was collected, and each year was weighted according to Japanese age-stratified population structure based on 5-year age group data [13]. We did not include cost of antibiotics assessed as appropriate. Costs of oral third-generation cephalosporins, macrolides, and fluoroquinolones were separately evaluated. We did not consider the cost of treatment for side effects caused by unnecessary antibiotic prescription (e.g. anaphylaxis, urticaria, and diarrhoea). We assumed that each patient’s diagnosis was appropriate and antibiotic prescription for URI did not treat any bacterial infection case that was misdiagnosed as URI.

To reflect uncertainty, we estimated 95% confidence intervals (CIs) of the proportion of population coverage, the proportion of URI cases prescribed antibiotics, and the cost of antibiotics in each case. We conducted the binomial test to estimate 95% CIs of population coverage and the proportion of antibiotic prescription. For the cost of antibiotics, we conducted the t-test to estimate 95% CIs.

All analyses were conducted by Stata MP15 [14] (for data aggregation) and R (for statistical analyses) version 3.5.2 [15]..

### Role of the funding source

The funder of the study had no role in study design, data collection, data analysis, data interpretation, or writing of the report. The first and corresponding author had full access to all the data in the study and had final responsibility for the decision to submit for publication.

## Results

### Study population

The JMDC database covered about 3% of the total population of Japan under the age of 65 years (2·85% in 2013 and 3·45% in 2016). Details of enrollees are described in Table 1.

**Table 1:** Number of enrollees in each age group^a^

Age group (years)	2013	2014	2015	2016
**0–4**	**300,045 (5·73)**	**349,827 (6·71)**	**364,043 (7·27)**	**378,095 (7·62)**
**5–9**	**186,265 (3·47)**	**199,763 (3·76)**	**190,513 (3·58)**	**183,465 (3·46)**
**10–14**	**178,937 (3·09)**	**195,988 (3·43)**	**185,914 (3·31)**	**178,224 (3·23)**
**15–19**	**181,308 (3·0)**	**232,657 (3·87)**	**229,207 (3·79)**	**229,102 (3·79)**
**20–24**	**206,669 (3·33)**	**301,604 (4·86)**	**310,845 (5·10)**	**319,438 (5·19)**
**25–29**	**234,889 (3·42)**	**301,904 (4·52)**	**301,886 (4·62)**	**303,908 (4·75)**
**30–34**	**254,486 (3·34)**	**304,273 (4·08)**	**298,905 (4·04)**	**297,681 (4·10)**
**35–39**	**279,942 (3·09)**	**319,855 (3·69)**	**309,508 (3·68)**	**302,943 (3·73)**
**40–44**	**266,656 (2·76)**	**299,446 (3·06)**	**288,814 (2·93)**	**281,933 (2·90)**
**45–49**	**219,357 (2·61)**	**242,943 (2·82)**	**233,439 (2·66)**	**227,467 (2·45)**
**50–54**	**174,821 (2·26)**	**198,847 (2·55)**	**190,321 (2·37)**	**184,999 (2·34)**
**55–59**	**129,055 (1·67)**	**172,490 (2·25)**	**164,890 (2·17)**	**159,649 (2·12)**
**60–64**	**107,417 (1·11)**	**146,718 (1·63)**	**142,367 (1·66)**	**138,287 (1·70)**
**Total**	**2,719,847 (2·85)**	**3,266,315 (3·47)**	**3,210,652 (3·44)**	**3,185,191 (3·45)**

^a^Values in parentheses represent the proportion of enrollees of total number of Japanese populations in each age group

### Proportion of antibiotic prescription for URI

The estimated proportion of inappropriate antibiotic prescription for URI in each year and age group is shown in Table 2. About 30% of URI cases in ambulatory care settings were prescribed antibiotics (32·41% in 2013 and 29·36% in 2016).

**Table 2:** Estimated percentage of inappropriate antibiotic prescription for URI in ambulatory care^a^

Age group (years)	2013	2014	2015	2016
**0–4**	**22·45 (22·37–22·52)**	**21**·**59 (21**·**52–21**·**66)**	**20**·**14 (20**·**07–20**·**21)**	**18**·**87 (18**·**80–18**·**94)**
**5–9**	**30·92 (30·79–31·05)**	**30**·**37 (30**·**25–30**·**50)**	**28**·**32 (28**·**20–28**·**44)**	**26**·**75 (26**·**63–26**·**87)**
**10–14**	**36·88 (36·69–37·08)**	**35**·**95 (35**·**76–36**·**13)**	**34**·**56 (34**·**39–34**·**74)**	**33**·**04 (32**·**86–33**·**22)**
**15–19**	**43·29 (43·0–43·58)**	**42**·**02 (41**·**75–42**·**30)**	**41**·**71 (41**·**45–41**·**97)**	**37**·**87 (37**·**63–38**·**12)**
**20–24**	**44·05 (43·74–44·36)**	**42**·**72 (42**·**42–43**·**01)**	**43**·**61 (43**·**32–43**·**91)**	**40**·**49 (40**·**21–40**·**78)**
**25–29**	**42·51 (42·24–42·78)**	**41**·**68 (41**·**42–41**·**94)**	**42**·**09 (41**·**84–42**·**35)**	**39**·**99 (39**·**73–40**·**24)**
**30–34**	**42·74 (42·51–42·96)**	**42**·**01 (41**·**79–42**·**23)**	**41**·**52 (41**·**30–41**·**74)**	**39**·**80 (39**·**58–40**·**02)**
**35–39**	**42·82 (42·61–43·04)**	**42**·**09 (41**·**88–42**·**30)**	**41**·**45 (41**·**24–41**·**66)**	**39**·**98 (39**·**77–40**·**19)**
**40–44**	**41·6 (41·37–41·82)**	**40**·**95 (40**·**74–41**·**17)**	**40**·**46 (40**·**26–40**·**67)**	**39**·**04 (38**·**83–39**·**25)**
**45–49**	**39**·**10 (38**·**85–39**·**35)**	**38**·**16 (37**·**93–38**·**40)**	**38**·**40 (38**·**17–38**·**62)**	**36**·**87 (36**·**65–37**·**10)**
**50–54**	**36**·**75 (36**·**49–37**·**01)**	**36**·**31 (36**·**06–36**·**56)**	**36**·**09 (35**·**85–36**·**33)**	**34**·**79 (34**·**55–35**·**02)**
**55–59**	**35**·**45 (35**·**16–35**·**74)**	**34**·**88 (34**·**60–35**·**15)**	**35**·**04 (34**·**78–35**·**30)**	**33**·**45 (33**·**20–33**·**71)**
**60–64**	**33**·**62 (33**·**30–33**·**94)**	**33**·**06 (32**·**74–33**·**38)**	**33**·**27 (32**·**96–33**·**58)**	**31**·**71 (31**·**40–32**·**02)**
**Total**	**32**·**41 (32**·**37–32**·**47)**	**31**·**68 (31**·**63–31**·**73)**	**31**·**0 (30**·**95–31**·**05)**	**29**·**36 (29**·**31–29**·**40)**

^a^Data are expressed as percentages. Values in parentheses represent 95% confidence intervals

### Cost of antibiotic prescription

The estimated additional cost of inappropriate antibiotic prescription for URI at the total Japanese population level is shown in Table 3. Total additional cost amounts were 300–400 million USD annually from the healthcare payer’s perspective (423·6 million USD in 2013 and 297·1 million USD in 2016).

**Table 3:** Estimated additional cost of antibiotic use for URI in ambulatory care (unit = million USD)^a^

Age group (years)	2013	2014	2015	2016
**0–4**	**40**·**3 (39**·**9–40**·**7)**	**32**·**7 (32**·**4–33**·**0)**	**27**·**2 (26**·**9–27**·**5)**	**22**·**1 (21**·**8–22**·**3)**
**5–9**	**56**·**6 (55**·**9–57**·**3)**	**50**·**1 (49**·**5–50**·**7)**	**51**·**1 (50**·**5–51**·**8)**	**43**·**4 (42**·**8–43**·**9)**
**10–14**	**31**·**2 (30**·**7–31**·**7)**	**27**·**7 (27**·**3–28**·**1)**	**29**·**0 (28**·**6–29**·**4)**	**24**·**4 (24**·**0–24**·**7)**
**15–19**	**19**·**5 (19**·**1–19**·**8)**	**14**·**6 (14**·**4–14**·**8)**	**16**·**1 (15**·**8–16**·**4)**	**14**·**0 (13**·**8–14**·**3)**
**20–24**	**17**·**2 (16**·**9–17**·**5)**	**11**·**4 (11**·**2–11**·**6)**	**11**·**1 (11**·**0–11**·**3)**	**9**·**5 (9**·**3–9**·**6)**
**25–29**	**22**·**4 (22**·**0–22**·**7)**	**16**·**1 (15**·**8–16**·**4)**	**15**·**5 (15**·**3–15**·**8)**	**12**·**6 (12**·**4–12**·**8)**
**30–34**	**31**·**2 (30**·**8–31**·**7)**	**23**·**9 (23**·**6–24**·**2)**	**23**·**9 (23**·**5–24**·**2)**	**19**·**8 (19**·**5–20**·**1)**
**35–39**	**38**·**0 (37**·**4–38**·**5)**	**29**·**9 (29**·**5–30**·**3)**	**29**·**6 (29**·**2–30**·**0)**	**23**·**7 (23**·**4–24**·**1)**
**40–44**	**39**·**7 (39**·**1–40**·**2)**	**34**·**9 (34**·**4–35**·**4)**	**37**·**6 (37**·**1–38**·**1)**	**30**·**5 (30**·**1–30**·**9)**
**45–49**	**31**·**9 (31**·**3–32**·**4)**	**28**·**4 (28**·**0–28**·**9)**	**31**·**8 (31**·**3–32**·**3)**	**30**·**3 (29**·**8–30**·**8)**
**50–54**	**29**·**4 (28**·**8–29**·**9)**	**26**·**2 (25**·**7–26**·**7)**	**30**·**0 (29**·**5–30**·**6)**	**26**·**2 (25**·**7–26**·**7)**
**55–59**	**31**·**1 (30**·**4–31**·**8)**	**23**·**0 (22**·**5–23**·**4)**	**25**·**3 (24**·**8–25**·**8)**	**23**·**0 (22**·**6–23**·**5)**
**60–64**	**35**·**4 (34**·**5–36**·**3)**	**22**·**0 (21**·**5–22**·**6)**	**21**·**7 (21**·**2–22**·**2)**	**17**·**7 (17**·**3–18**·**2)**
**Total**	**423**·**6 (416**·**8–430**·**5)**	**340**·**9 (335**·**7–346**·**2)**	**349**·**9 (344**·**5–355**·**3)**	**297**·**1 (292**·**4–301**·**9)**

^a^Values in parentheses represent 95% confidence intervals

**Fig. 1:**
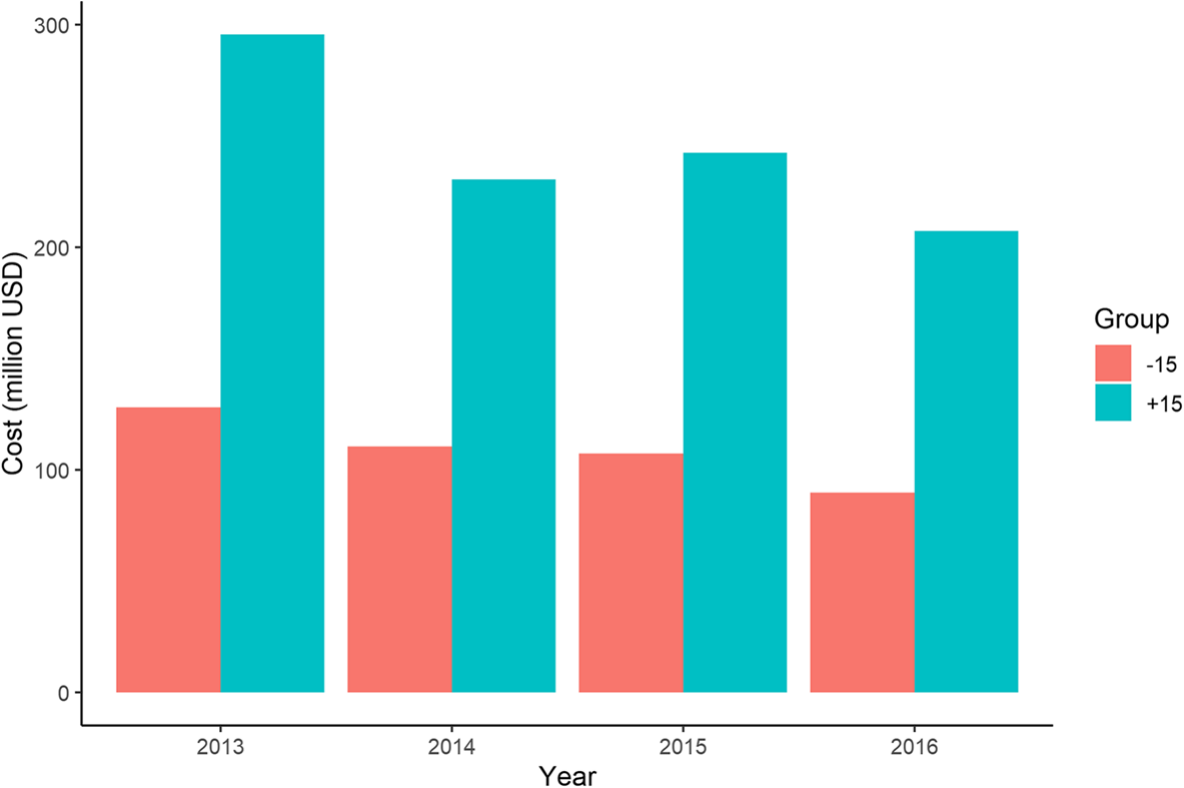
Additional cost of antibiotic prescription for URI in children and adults

Figure 1 describes the breakdown of the additional cost of antibiotic prescriptions by age group. About 30% of all antibiotic prescriptions for URI were for children under 15 years (30·2% in both 2013 and 2016).

### Breakdown of antibiotic class

**Fig. 2:**
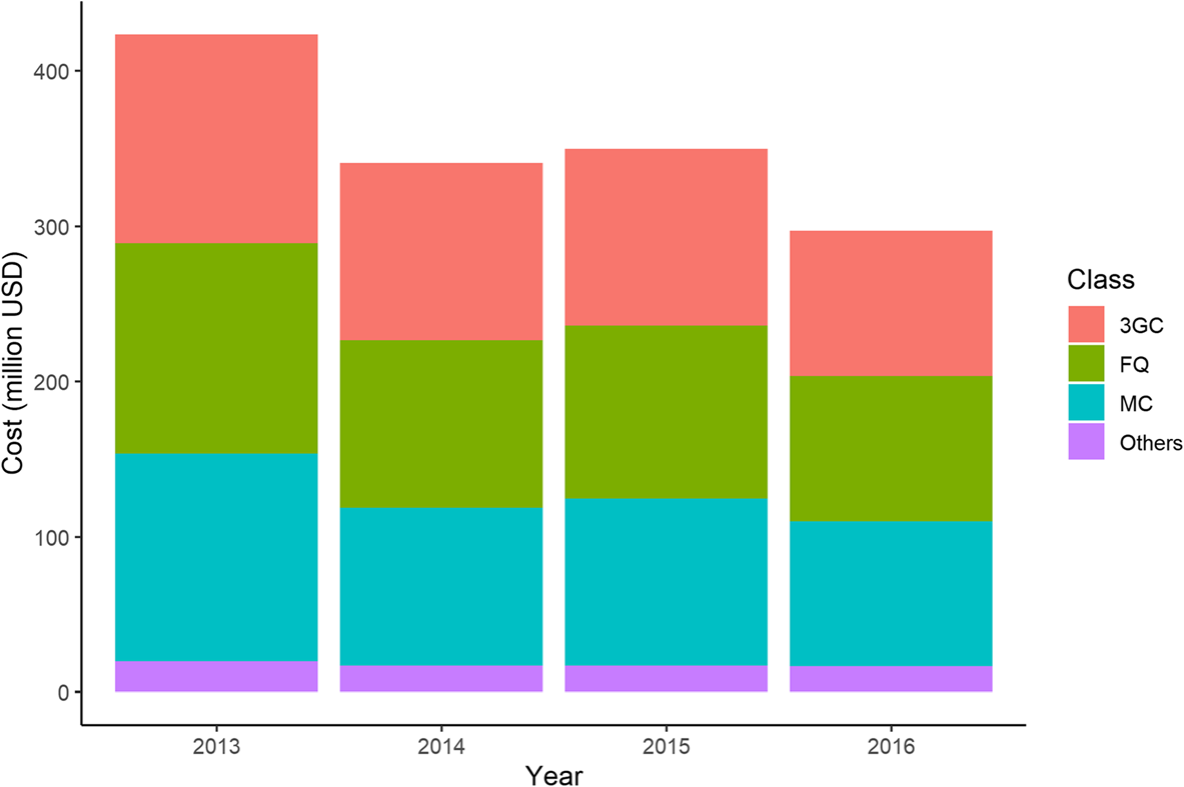
Breakdown of annual additional cost of antibiotic prescription by antibiotic class. 3GC, third-generation cephalosporins; FQ, fluoroquinolones; MC, macrolides

Annual estimated percentages and additional costs of antibiotic prescription for three broad-spectrum antibiotic classes (third-generation cephalosporins, macrolides, and fluoroquinolones) are shown in Fig. 2 and Tables 4 and 5. Third-generation cephalosporins were prescribed to about 12% of URI cases (13·17% in 2013 and 11·33% in 2016). Macrolides were prescribed to about 10% of URI cases (11·06% in 2013 and 10·38% in 2016) and fluoroquinolones were prescribed to about 5% of URI cases (4·60% in 2013 and 4·23% in 2016). Additional prescription costs of these three antibiotic classes amounted to 403·9 million USD in 2013 and 280·0 million USD in 2016. Details of antibiotics prescribed for URI by class are available in Supplementary file 3.

**Table 4:** Estimated percentages of broad-spectrum antibiotic prescription in ambulatory care^a^

Class	2013	2014	2015	2016
**Third-generation cephalosporins**	**13**·**17** **(13**·**14–13**·**21)**	**13**·**0** **(12**·**97–13**·**04)**	**12**·**41** **(12**·**38–12**·**44)**	**11**·**33** **(11**·**29–11**·**36)**
**Macrolides**	**11**·**06** **(11**·**02–11**·**09)**	**10**·**56** **(10**·**53–10**·**59)**	**10**·**58** **(10**·**55–10**·**61)**	**10**·**38** **(10**·**35–10**·**41)**
**Fluoroquinolones**	**4**·**60 (4**·**58–4**·**62)**	**4**·**45 (4**·**43–4**·**47)**	**4**·**55 (4**·**53–4**·**57)**	**4**·**23 (4**·**21–4**·**26)**

^a^Values in parentheses represent 95% confidence intervals

**Table 5:** Estimated additional costs of broad-spectrum antibiotic prescription in ambulatory care (unit = million USD)^a^

Class	2013	2014	2015	2016
**Third-generation cephalosporins**	**134·7** **(132·0–137·5)**	**114·1** **(111·9–116·3)**	**113·8** **(111·5–116·1)**	**93·7** **(91·7–95·7)**
**Macrolides**	**133·8** **(130·6–137·1)**	**101·7** **(99·3–104·1)**	**108·1** **(105·6–110·5)**	**93·4** **(91·2–95·6)**
**Fluoroquinolones**	**135·4** **(131·2–139·7)**	**108·1** **(104·9–111·4)**	**111·2** **(107·9–114·6)**	**93·5** **(90·6–96·5)**
**Total**	**403·9** **(394·8–414·3)**	**323·9** **(316·1–331·8)**	**333·1** **(325·0–341·2)**	**280·0** **(273·5–287·8)**

^a^ Values in parentheses represent 95% confidence intervals

We also evaluated the share of broad-spectrum antibiotics in the total annual cost of antibiotic prescription. Three antibiotic classes (third-generation cephalosporins, macrolides, and fluoroquinolones) accounted for over 90% of total additional antibiotic prescription costs for URI (95·3% in 2013 and 94·2% in 2016).

## Discussion

We developed a national population level cost estimation about the unnecessary cost of antibiotic prescription for URI. To our knowledge, the present study is the first to assess physicians’ antibiotic prescription behaviour not only in view of the proportion of antibiotic prescriptions but also the associated additional costs. By adding the viewpoint of cost, our findings show novel characteristics of inappropriate antibiotic prescription in ambulatory care settings.

First, both the proportion and cost of unnecessary antibiotic prescription for URI have decreased in recent years. The proportion of URI cases to which physicians prescribed antibiotics was 32·41% in 2013 but only 29·36% in 2016. The cost of antibiotic prescriptions for URI cases was 423·6 million USD in 2013 and decreased to 297·1 million USD in 2016. The proportion of URI cases to which physicians prescribed unnecessary antibiotics demonstrated a gradually decreasing trend every year and the total annual cost of antibiotic prescriptions for URI in 2016 was about 30% lower than that in 2013. According to a previous study, Japanese physicians had prescribed antibiotics for 60% of URI cases in 2005 [7]. It is difficult to compare the results of the present study and that of the previous study because of sample size (the previous study analysed only 2577 claims), sampling period (the previous study collected claims from January to March 2005 only), and other factors. Nevertheless, this decreasing trend of unnecessary antibiotic use might have already existed in Japan early in this century.

As mentioned above, the Japanese government established the National Action Plan on AMR in 2016 [6]. While it will take a few more years to assess the impact of this plan because it is newly established, our findings might reflect physicians’ alteration in awareness on AMR rather than behavioural change in antibiotic prescription.

Conversely, the proportion of antibiotic prescriptions for URI is difficult to compare with previous studies from foreign countries due to differences in settings and conditions. For example, Barnett and Linder reported that the antibiotic prescribing rate dropped from roughly 80 to 70% around 1993 and decreased again around 2000 to 60% [16]. However, their study was limited to adults and ‘sore throat’ cases; therefore, a large number of bacterial pharyngitis cases were likely included. Finkelstein and colleagues examined the effect of an educational outreach intervention in ambulatory care but only evaluated paediatric cases [17]. Fleming-Dutra and colleagues conducted a more extensive analysis, however, their focus was on whole ambulatory care, which includes diagnoses sometimes appropriate for antibiotic prescription (e.g. otitis media and sinusitis).

Furthermore, we must take the differences in healthcare system between Japan and other countries into consideration. The Japanese healthcare system generally secures ‘free access’ to healthcare services, regardless of facility level, and there is no limitation on the frequency of ambulatory care visit [18]. This implies that a larger number of URI patients tends to visit ambulatory care although URI is basically a self-limited disease and there are substantial numbers of URI patients amongst patients admitted to healthcare facilities [19, 20]. This fact makes it more difficult to compare the proportion of URI cases prescribed antibiotics prescribed. While decreasing trends in the proportion and cost of antibiotic prescription for URI are favourable findings, the main driver of this trend and the current situation of Japanese ambulatory care in global society are still not clear.

Second, our results demonstrated the importance of appropriate antibiotic use, especially in paediatric ambulatory care. Despite the comparatively low rate of antibiotic prescription in children and the ratio of children to the total population, the additional cost in children under 15 years accounted for over 30% of total additional costs annually. This finding likely reflects the frequency of healthcare facility visits attributed to URI by children [21]. Additionally, most Japanese nursery schools and kindergartens require caregivers to bring their children to healthcare facilities when they catch a cold [22]. As a result, children are exposed to the risk of unnecessary antibiotic prescription more frequently than adults are in Japanese ambulatory care. Therefore, children should be an appropriate target population for interventions to promote appropriate antibiotic use.

Third, it is noteworthy that broad-spectrum oral antibiotics accounted for the majority of additional antibiotic prescription costs for URI. Three antibiotic classes (third-generation cephalosporins, macrolides, and fluoroquinolones) comprised almost 95% of total additional antibiotic prescription costs for URI in Japan. Our finding of a high prescription rate of third-generation cephalosporins and macrolides is compatible with findings from a previous study [23]. The fluoroquinolone prescription rate was comparatively low, however, it can be attributed to the clinical contraindication of fluoroquinolones for children. We observed an extremely low prescription rate of fluoroquinolones in children under 5 years and a high rate in adults (Supplementary file 3). Considering these findings with the difference in drug prices between these broad-spectrum antibiotics and other narrow-spectrum antibiotics (e.g. penicillins), we can understand the significance of appropriate use of broad-spectrum antibiotics more profoundly. Compared with other high-income countries, Japan demonstrated higher consumption rates of these three antibiotic classes [24]. Namely, these broad-spectrum antibiotics could be an appropriate target for antimicrobial stewardship in ambulatory care in Japan, not only from the standpoint of antimicrobial resistance but also that of economic burden.

### Strengths and limitations

A major strength of the present study is the use of a large number of individual patient-level claims data, which cover both children and adults. Conversely, our data did not include claims of patients 65 years of age or older.

While millions of individual claims are included in the dataset, its representativeness is not completely assured because our data are based on information from health insurance purchased by enterprises. Nevertheless, a low proportion of self-employed people (8·48% in 2017) and low unemployment rate (3·1% in 2016) in Japan [25] enable us to justify the result of our analyses. Because self-employed and unemployed people are also covered by other types of national health insurance in the Japanese healthcare system and out-of-pocket costs are reimbursed according to each individual’s income, we can expect that healthcare-seeking behaviour would not be greatly different among employed, self-employed, and unemployed individuals. Nonetheless, further study would be desirable to examine the difference in healthcare-seeking behaviour brought by employment status.

In addition, diagnoses in the dataset are sometimes unreliable. It is often the case that physicians make different diagnosis in the claims on purpose to justify their examinations and prescriptions. Nevertheless, physicians never make a fake diagnosis of “URI” when they would like to prescribe antibiotics, but make a diagnosis of other bacterial infections in order to justify their antibiotic prescriptions. Then therefore unreliable diagnoses might not overemphasize the cost of inappropriate antibiotic prescription.

Another strength is that we introduced the concept of cost. Although the rate of antibiotic prescription or defined daily dose [26] are indicators understood intuitively, our findings added another aspect of broad-spectrum antibiotics. As one important limitation, our present analyses showed conservative results because we did not consider any additional cost of adverse effects of antibiotics. For example, rush, diarrhoea, and anaphylaxis are general adverse effects sometimes observed with systemic use of oral antibiotics. Of course, these adverse events might impose additional medical costs, however, there is no appropriate information about costs and the probability of such events in Japan thus far. Furthermore, broad-spectrum antibiotics have their own adverse effects. For example, oral third-generation cephalosporins can cause hypocarnitinaemia on rare occasions [27]. Azithromycin is associated with a slightly increased risk of cardiovascular death [28]. Fluoroquinolones uncommonly cause tendon rupture [29] and QT prolongation [30] and the US Food and Drug Administration updated its drug safety information for fluoroquinolone due to the rare but serious risk of aortic ruptures or tears in certain patient [31]. If we can take these adverse events into consideration as a form of medical cost, then the estimated economic burden of antibiotic prescription for URI might be more precise.

## Conclusions

As we have shown, the present study gives an extensive understanding about the impact of inappropriate antibiotic prescription for URI. Although a decreasing trend was observed, the annual additional cost of antibiotic prescription for URI could be considered a substantial economic burden in Japan. Our study also suggests that appropriate use of broad-spectrum oral antibiotics might be critical to reduce unnecessary medical costs in Japanese ambulatory care.

## Supplementary information

**Additional file 1.** **Supplementary Spreadsheet**. Details of the discussion process to judge appropriateness of antibiotic prescription.

**Additional file 2. Supplementary Table**. Appropriateness of antibiotic prescribing for ARTI used in the study.

**Additional file 3. Table S1.** Estimated percentage of third-generation cephalosporin prescriptions for URI in ambulatory care. **Table S2.** Estimated additional cost of third-generation cephalosporin use for URI in ambulatory care. **Table S3.** Estimated percentage of macrolide prescriptions for URI in ambulatory care. **Table S4.** Estimated additional cost of macrolide use for URI in ambulatory care. **Table S5.** Estimated percentage of fluoroquinolone prescriptions for URI in ambulatory care. **Table S6.** Estimated additional cost of fluoroquinolone use for URI in ambulatory care.

## Data Availability

The datasets for the study are not publicly available due to company policy of JMDC Inc. Data are, however, available from the corresponding author upon reasonable request and with the permission of the JMDC Inc.

## Abbreviations

3GC: Third-generation cephalosporins
AMR: Antimicrobial resistance
CI: Confidence interval
FQ: Fluoroquinolones
ICD-10: International Classification of Diseases, 10th Revision
JPY: Japanese yen
MC: Macrolides
URI: Upper respiratory infection
USD: United States dollar
