# Supplementary material for: Cost of inappropriate antimicrobial use for upper respiratory infection in Japan
Source: BMC Health Serv Res. 2020 Feb 28;20:153. doi: 10.1186/s12913-020-5021-1 (PMC7048145; doi:10.1186/s12913-020-5021-1)
Supplement: Supplementary file 2 — Additional file 2. Supplementary Table. Appropriateness of antibiotic prescribing for ARTI used in the study. [file 12913_2020_5021_MOESM2_ESM.docx]

**Supplementary Table. Appropriateness of antibiotic prescribing for ARTI used in the study**

| ICD10 codes | | | Diagnosis in Japanese Claims Code | Appropriateness |
| --- | --- | --- | --- | --- |
| J00 | Acute nasopharyngitis [common cold] | | | |
|  | J00 | Acute nasopharyngitis [common cold] | Acute rhinitis | Not appropriate |
|  |  |  | Common cold | Not appropriate |
|  |  |  | Infective nasopharyngitis | Not appropriate |
|  |  |  | Infective rhinitis | Not appropriate |
|  |  |  | Nasopharyngitis | Not appropriate |
| J01 | Acute sinusitis | | | |
|  | J010 | Acute maxillary sinusitis | Acute maxillary sinusitis | Appropriate |
|  | J011 | Acute frontal sinusitis | Acute frontal sinusitis | Appropriate |
|  | J012 | Acute ethmoidal sinusitis | Acute ethmoidal sinusitis | Appropriate |
|  | J013 | Acute sphenoidal sinusitis | Acute sphenoidal sinusitis | Appropriate |
|  | J014 | Acute pansinusitis | Acute pansinusitis | Appropriate |
|  | J019 | Acute sinusitis, unspecified | Acute sinusitis, unspecified | Appropriate |
| J02 | Acute pharyngitis | | | |
|  | J020 | Streptococcal pharyngitis | Septic pharyngitis | Appropriate |
|  |  |  | Streptococcal angina | Appropriate |
|  |  |  | Streptococcal sore throat | Appropriate |
|  | J028 | Acute pharyngitis due to other specified organisms | Adenoviral pharyngitis | Not appropriate |
|  |  |  | Haemophilus influenzae pharyngitis | Appropriate |
|  |  |  | Membranous pharyngitis | Not appropriate |
|  |  |  | Pneumococcal pharyngitis | Appropriate |
|  |  |  | Pseudomembranous pharyngitis | Not appropriate |
|  |  |  | Staphylococcal pharyngitis | Not appropriate |
|  |  |  | Viral pharyngitis | Not appropriate |
|  | J029 | Acute pharyngitis, unspecified | Acute pharyngitis | Not appropriate |
|  |  |  | Acute suppurative pharyngitis | Appropriate |
|  |  |  | Angina | Not appropriate |
|  |  |  | Catarrhal pharyngitis | Not appropriate |
|  |  |  | Gangrenous pharyngitis | Appropriate |
|  |  |  | Infective pharyngitis | Not appropriate |
|  |  |  | Lower Pharyngitis | Not appropriate |
|  |  |  | Pharyngitis | Not appropriate |
|  |  |  | Sore throat | Not appropriate |
|  |  |  | Ulcerative pharyngitis | Not appropriate |
|  |  |  | Upper Pharyngitis | Not appropriate |
| J03 | Acute tonsillitis | | | |
|  | J030 | Streptococcal tonsillitis | Streptococcal tonsillitis | Appropriate |
|  | J038 | Acute tonsillitis due to other specified organisms | Adenoviral tonsillitis | Not appropriate |
|  |  |  | Staphylococcal tonsillitis | Not appropriate |
|  |  |  | Viral tonsillitis | Not appropriate |
|  | J039 | Acute tonsillitis, unspecified | Acute adenoiditis pharyngitis | Not appropriate |
|  |  |  | Acute adenoiditis tonsillitis | Not appropriate |
|  |  |  | Acute gangrenous tonsillitis | Appropriate |
|  |  |  | Acute lacunar tonsillitis | Not appropriate |
|  |  |  | Acute suppurative tonsillitis | Appropriate |
|  |  |  | Acute tonsillitis | Not appropriate |
|  |  |  | Acute ulcerative tonsillitis | Not appropriate |
|  |  |  | Angina tonsillitis | Not appropriate |
|  |  |  | Habitual angina | Not appropriate |
|  |  |  | Habitual tonsillitis | Not appropriate |
|  |  |  | Lacunar angina | Not appropriate |
|  |  |  | Lingual tonsillitis | Not appropriate |
|  |  |  | Pseudomembranous tonsillitis | Not appropriate |
|  |  |  | Tonsillitis | Not appropriate |
| J04 | Acute laryngitis and tracheitis | | | |
|  | J040 | Acute laryngitis | Acute edematous laryngitis | Not appropriate |
|  |  |  | Acute gangrenous tonsillitis laryngitis | Appropriate |
|  |  |  | Acute glottitis | Not appropriate |
|  |  |  | Acute laryngitis | Not appropriate |
|  |  |  | Acute streptococcal laryngitis | Appropriate |
|  |  |  | Acute subglottic laryngitis | Not appropriate |
|  |  |  | Acute ulcerative laryngitis | Not appropriate |
|  |  |  | Hemophilus influenzae laryngitis | Appropriate |
|  |  |  | Laryngitis | Not appropriate |
|  |  |  | Perilaryngitis | Not appropriate |
|  |  |  | Pseudomembranous pseudomembranous | Not appropriate |
|  |  |  | Suppurative laryngitis | Appropriate |
|  | J041 | Acute tracheitis | Acute catarrhal tracheitis | Not appropriate |
|  |  |  | Acute tracheitis | Not appropriate |
|  |  |  | Hemorrhagic tracheitis | Not appropriate |
|  |  |  | Streptococcal tracheitis | Appropriate |
|  |  |  | Viral tracheitis | Not appropriate |
|  | J042 | Acute laryngotracheitis | Acute laryngotracheitis | Not appropriate |
|  |  |  | Haemophilus influenzae laryngotracheitis | Appropriate |
|  |  |  | Infective laryngotracheitis | Appropriate |
|  |  |  | Streptococcal laryngotracheitis | Appropriate |
| J05 | Acute obstructive laryngitis [croup] and epiglottitis | | | |
|  | J050 | Acute obstructive laryngitis [croup] | Acute obstructive laryngitis | Not appropriate |
|  |  |  | Acute spasmodic laryngotracheitis | Not appropriate |
|  | J051 | Acute epiglottitis | Acute epiglottitis | Appropriate |
|  |  |  | Epiglottitis | Appropriate |
| J06 | Acute upper respiratory infections of multiple and unspecified sites | | | |
|  | J060 | Acute laryngopharyngitis | Acute laryngopharyngitis | Not appropriate |
|  |  |  | Laryngopharyngitis | Not appropriate |
|  | J068 | Other acute upper respiratory infections of multiple sites | Acue laryngotonsillitis | Not appropriate |
|  |  |  | Acute tonsillitis palatina | Not appropriate |
|  |  |  | Laryngotonsillitis | Not appropriate |
|  |  |  | Laryngotracheitis | Not appropriate |
|  | J069 | Acute upper respiratory infection, unspecified | Acute upper respiratory infection | Not appropriate |
|  |  |  | Streptococcal upper respiratory infection | Appropriate |
| J20 | Acute bronchitis | | | |
|  | J200 | Acute bronchitis due to Mycoplasma pneumoniae | Acute bronchitis due to Mycoplasma pneumoniae | Appropriate |
|  | J201 | Acute bronchitis due to Haemophilus influenzae | Acute bronchitis due to Haemophilus influenzae | Appropriate |
|  | J202 | Acute bronchitis due to streptococcus | Acute bronchitis due to streptococcus | Appropriate |
|  | J203 | Acute bronchitis due to coxsackievirus | Acute bronchitis due to coxsackievirus | Not appropriate |
|  | J204 | Acute bronchitis due to parainfluenza virus | Acute bronchitis due to parainfluenza virus | Not appropriate |
|  | J205 | Acute bronchitis due to respiratory syncytial virus | Acute bronchitis due to respiratory syncytial virus | Not appropriate |
|  | J206 | Acute bronchitis due to rhinovirus | Acute bronchitis due to rhinovirus | Not appropriate |
|  | J207 | Acute bronchitis due to echovirus | Acute bronchitis due to echovirus | Not appropriate |
|  | J208 | Acute bronchitis due to other specified organisms | Acute bronchitis due to human metapneumovirus bronchitis | Not appropriate |
|  |  |  | Viral bronchitis | Not appropriate |
|  | J209 | Acute bronchitis, unspecified | Acute bronchitis | Not appropriate |
|  |  |  | Acute laryngitis tracheobronchitis | Not appropriate |
|  |  |  | Acute recurrent bronchitis | Not appropriate |
|  |  |  | Acute tracheobronchitis | Not appropriate |
|  |  |  | Croupous bronchitis | Not appropriate |
|  |  |  | Exudative bronchitis | Not appropriate |
|  |  |  | Pseudomembranous bronchitis | Not appropriate |
|  |  |  | Septic bronchitis | Appropriate |
|  |  |  | Subacute bronchitis | Not appropriate |
| J21 | Acute bronchiolitis | | | |
|  | J210 | Acute bronchiolitis due to respiratory syncytial virus | Acute bronchiolitis due to respiratory syncytial virus | Not appropriate |
|  | J218 | Acute bronchiolitis due to other specified organisms | Acute bronchiolitis due to Human metapneumovirus | Not appropriate |
|  | J219 | Acute bronchiolitis, unspecified | Acute bronchiolitis | Not appropriate |
| J22 | Unspecified acute lower respiratory infection | | | |
|  | J22 | Unspecified acute lower respiratory infection | Acute (lower) respiratory (tract) infection | Not appropriate |
