# Supplementary material for: Cost of inappropriate antimicrobial use for upper respiratory infection in Japan
Source: BMC Health Serv Res. 2020 Feb 28;20:153. doi: 10.1186/s12913-020-5021-1 (PMC7048145; doi:10.1186/s12913-020-5021-1)
Supplement: Supplementary file 3 — Additional file 3. Table S1. Estimated percentage of third-generation cephalosporin prescriptions for URI in ambulatory care. Table S2. Estimated additional cost of third-generation cephalosporin use for URI in ambulatory care. Table S3. Estimated percentage of macrolide prescriptions for URI in ambulatory care. Table S4. Estimated additional cost of macrolide use for URI in ambulatory care. Table S5. Estimated percentage of fluoroquinolone prescriptions for URI in ambulatory care. Table S6. Estimated additional cost of fluoroquinolone use for URI in ambulatory care. [file 12913_2020_5021_MOESM3_ESM.docx]

**Cost of antimicrobial use for upper respiratory infection in Japan**

**Supplementary file 3**

Shinya Tsuzuki*, Yuki Kimura, Masahiro Ishikane, Yoshiki Kusama, and Norio Ohmagari

Contents

**Table S1: Estimated percentage of third-generation cephalosporin prescriptions for URI in ambulatory care**

**Table S2: Estimated additional cost of third-generation cephalosporin use for URI in ambulatory care**

**Table S3: Estimated percentage of macrolide prescriptions for URI in ambulatory care**

**Table S4: Estimated additional cost of macrolide use for URI in ambulatory care**

**Table S5: Estimated percentage of fluoroquinolone prescriptions for URI in ambulatory care**

**Table S6: Estimated additional cost of fluoroquinolone use for URI in ambulatory care**

**Table S1. Estimated percentage of third-generation cephalosporin prescriptions for URI in ambulatory care***

| **Age group (years)** | **2013** | **2014** | **2015** | **2016** |
| --- | --- | --- | --- | --- |
| 0–4 | 10·78 (10·73–10·84) | 10·39 (10·34–10·44) | 9·63 (9·58–9·69) | 8·67 (8·62–8·72) |
| 5–9 | 14·82 (14·72–14·92) | 15·01 (14·91–15·11) | 13·44 (13·35–13·53) | 11·99 (11·90–12·07) |
| 10–14 | 16·89 (16·74–17·05) | 16·85 (16·70–16·99) | 15·38 (15·24–15·52) | 14·19 (14·06–14·33) |
| 15–19 | 18·04 (17·81–18·26) | 17·81 (17·60–18·02) | 17·34 (17·14–17·55) | 14·93 (14·75–15·12) |
| 20–24 | 16·06 (15·83–16·29) | 15·84 (15·62–16·06) | 15·88 (15·66–16·10) | 14·66 (14·46–14·86) |
| 25–29 | 15·40 (15·21–15·60) | 15·28 (15·10–15·47) | 15·42 (15·23–15·60) | 14·22 (14·03–14·40) |
| 30–34 | 15·04 (14·87–15·21) | 14·93 (14·76–15·09) | 14·84 (14·68–15·0) | 13·84 (13·69–14·0) |
| 35–39 | 14·58 (14·42–14·73) | 14·41 (14·26–14·56) | 14·07 (13·92–14·22) | 13·33 (13·18–13·48) |
| 40–44 | 13·63 (13·47–13·78) | 13·46 (13·31–13·61) | 13·23 (13·09–13·38) | 12·45 (12·31–12·59) |
| 45–49 | 12·70 (12·53–12·86) | 12·39 (12·23–12·55) | 12·40 (12·24–12·55) | 11·64 (11·49–11·79) |
| 50–54 | 11·95 (11·78–12·13) | 11·65 (11·49–11·82) | 11·65 (11·49–11·81) | 10·93 (10·78–11·08) |
| 55–59 | 11·22 (11·03–11·41) | 11·35 (11·17–11·54) | 11·21 (11·04–11·39) | 10·46 (10·30–10·63) |
| 60–64 | 10·49 (10·28–10·70) | 10·33 (10·13–10·54) | 10·33 (10·13–10·53) | 9·53 (9·33–9·73) |
| Total | 13·17 (13·14–13·21) | 13·0 (12·97–13·04) | 12·41 (12·38–12·44) | 11·33 (11·29–11·36) |

*Data are expressed in percentage. Values in parentheses represent 95% confidence intervals.

**Table S2. Estimated additional cost of third-generation cephalosporin use for URI in ambulatory care (in million USD)***

| **Age group (year)** | **2013** | **2014** | **2015** | **2016** |
| --- | --- | --- | --- | --- |
| 0–4 | 23·0 (22·8–23·3) | 19·1 (18·8–19·3) | 15·9 (15·7–15·9) | 12·3 (12·1–12·4) |
| 5–9 | 29·2 (28·8–29·7) | 27·3 (26·9–27·7) | 26·7 (26·3–27·2) | 21·5 (21·2–21·9) |
| 10–14 | 12·8 (12·5–13·1) | 12·0 (11·7–12·2) | 11·8 (11·6–12·0) | 9·8 (9·6–10·0) |
| 15–19 | 6·3 (6·2–6·5) | 5·1 (4·9–5·2) | 5·4 (5·3–5·6) | 4·6 (4·5–4·7) |
| 20–24 | 4·4 (4·3–4·5) | 3·0 (2·9–3·1) | 2·9 (2·9–3·0) | 2·5 (2·5–2·6) |
| 25–29 | 5·6 (5·5–5·7) | 4·1 (4·0–4·2) | 4·1 (4·0–4·2) | 3·3 (3·2–3·4) |
| 30–34 | 7·6 (7·4–7·8) | 6·0 (5·9–6·1) | 6·2 (6·0–6·3) | 5·0 (4·9–5·1) |
| 35–39 | 8·8 (8·7–9·0) | 7·2 (7·0–7·3) | 7·2 (7·0–7·3) | 5·7 (5·6–5·9) |
| 40–44 | 8·8 (8·7–9·0) | 8·0 (7·8–8·2) | 8·7 (8·5–8·9) | 7·0 (6·9–7·2) |
| 45–49 | 7·1 (6·9–7·3) | 6·4 (6·3–6·6) | 7·3 (7·1–7·4) | 6·9 (6·7–7·1) |
| 50–54 | 6·6 (6·4–6·8) | 5·8 (5·7–6·0) | 6·9 (6·7–7·0) | 5·9 (5·8–6·1) |
| 55–59 | 6·9 (6·6–7·1) | 5·3 (5·1–5·4) | 5·8 (5·6–5·9) | 5·2 (5·1–5·4) |
| 60–64 | 7·5 (7·2–7·8) | 4·9 (4·7–5·1) | 4·8 (4·7–5·0) | 3·9 (3·7–4·0) |
| Total | 134·7 (132·0–137·5) | 114·1 (111·9–116·3) | 113·8 (111·5–116·1) | 93·7 (91·7–95·7) |

*Values in parentheses represent 95% confidence intervals.

**Table S3. Estimated percentage of macrolide prescriptions for URI in ambulatory care***

| **Age group (year)** | **2013** | **2014** | **2015** | **2016** |
| --- | --- | --- | --- | --- |
| 0–4 | 7·01 (6·96–7·05) | 6·38 (6·34–6·43) | 6·04 (6·0–6·08) | 5·94 (5·90–5·98) |
| 5–9 | 12·25 (12·16–12·34) | 11·39 (11·30–11·48) | 11·22 (11·13–11·30) | 11·12 (11·04–11·21) |
| 10–14 | 15·42 (15·27–15·57) | 14·66 (14·53–14·80) | 14·82 (14·69–14·96) | 14·58 (14·44–14·71) |
| 15–19 | 14·84 (14·64–15·05) | 14·51 (14·32–14·71) | 14·62 (14·43–14·81) | 14·05 (13·87–14·23) |
| 20–24 | 13·27 (13·05–13·48) | 12·74 (12·54–12·95) | 13·11 (12·91–13·32) | 12·55 (12·36–12·74) |
| 25–29 | 12·71 (12·53–12·89) | 12·27 (12·10–12·44) | 12·56 (12·39–12·74) | 12·22 (12·05–12·40) |
| 30–34 | 13·46 (13·30–13·62) | 13·29 (13·13–13·44) | 13·05 (12·90–13·20) | 12·77 (12·62–12·92) |
| 35–39 | 14·03 (13·88–14·19) | 13·80 (13·65–13·95) | 13·73 (13·59–13·88) | 13·38 (13·23–13·53) |
| 40–44 | 13·95 (13·79–14·10) | 13·76 (13·61–13·91) | 13·67 (13·53–13·81) | 13·59 (13·45–13·74) |
| 45–49 | 13·39 (13·22–13·57) | 13·12 (12·95–13·28) | 13·32 (13·16–13·47) | 13·06 (12·91–13·22) |
| 50–54 | 12·51 (12·33–12·69) | 12·42 (12·25–12·59) | 12·41 (12·25–12·57) | 12·48 (12·32–12·64) |
| 55–59 | 12·12 (11·92–12·32) | 11·65 (11·46–11·83) | 12·14 (11·96–12·33) | 11·89 (11·71–12·06) |
| 60–64 | 11·69 (11·47–11·91) | 11·51 (11·30–11·73) | 11·54 (11·33–11·76) | 11·54 (11·33–11·75) |
| Total | 11·06 (11·02–11·09) | 10·56 (10·53–10·59) | 10·58 (10·55–10·61) | 10·38 (10·35–10·41) |

*Data are expressed in percentage. Values in parentheses represent 95% confidence intervals.

**Table S4. Estimated additional cost of macrolide use for URI in ambulatory care (in million USD)***

| **Age group (year)** | **2013** | **2014** | **2015** | **2016** |
| --- | --- | --- | --- | --- |
| 0–4 | 11·7 (11·5–11·9) | 8·7 (8·6–8·8) | 7·3 (7·2–7·4) | 6·3 (6·2–6·4) |
| 5–9 | 22·5 (22·1–22·9) | 18·2 (17·9–18·6) | 19·7 (19·3–20·0) | 17·4 (17·1–17·7) |
| 10–14 | 15·1 (14·7–15·4) | 12·7 (12·4–12·9) | 14·0 (13·7–14·2) | 11·8 (11·5–12·0) |
| 15–19 | 6·5 (6·3–6·7) | 4·7 (4·6–4·9) | 5·2 (5·1–5·4) | 4·7 (4·6–4·8) |
| 20–24 | 4·6 (4·5–4·7) | 2·8 (2·8–2·9) | 2·9 (2·8–2·9) | 2·4 (2·4–2·5) |
| 25–29 | 5·8 (5·7–6·0) | 3·9 (3·8–4·0) | 3·9 (3·8–4·0) | 3·2 (3·1–3·3) |
| 30–34 | 8·7 (8·5–8·9) | 6·2 (6·1–6·4) | 6·2 (6·1–6·4) | 5·2 (5·1–5·3) |
| 35–39 | 10·8 (10·6–11·1) | 8·0 (7·8–8·1) | 8·1 (8·0–8·3) | 6·4 (6·3–6·6) |
| 40–44 | 11·3 (11·1–11·6) | 9·5 (9·2–9·7) | 10·4 (10·2–10·6) | 8·5 (8·3–8·6) |
| 45–49 | 9·3 (9·0–9·5) | 7·8 (7·6–8·0) | 9·0 (8·8–9·2) | 8·6 (8·3–8·8) |
| 50–54 | 8·4 (8·2–8·7) | 7·0 (6·9–7·2) | 8·4 (8·2–8·6) | 7·5 (7·3–7·7) |
| 55–59 | 8·8 (8·6–9·1) | 6·1 (5·9–6·3) | 7·0 (6·8–7·2) | 6·4 (6·2–6·6) |
| 60–64 | 10·2 (9·8–10·6) | 6·1 (5·9–6·3) | 6·0 (5·8–6·2) | 5·0 (4·9–5·2) |
| Total | 133·8 (130·6–137·1) | 101·7 (99·3–104·1) | 108·1 (105·6–110·5) | 93·4 (91·2–95·6) |

*Values in parentheses represent 95% confidence intervals.

**Table S5. Estimated percentage of fluoroquinolone prescriptions for URI in ambulatory care***

| **Age group (year)** | **2013** | **2014** | **2015** | **2016** |
| --- | --- | --- | --- | --- |
| 0–4 | 0·20 (0·19–0·20) | 0·16 (0·16–0·17) | 0·13 (0·13–0·14) | 0·14 (0·13–0·14) |
| 5–9 | 0·34 (0·33–0·36) | 0·33 (0·31–0·35) | 0·34 (0·33–0·36) | 0·32 (0·31–0·34) |
| 10–14 | 1·53 (1·48–1·58) | 1·48 (1·43–1·53) | 1·45 (1·40–1·49) | 1·30 (1·26–1·34) |
| 15–19 | 7·10 (6·95–7·25) | 6·39 (6·26–6·53) | 6·54 (6·41–6·67) | 5·74 (5·62–5·85) |
| 20–24 | 11·22 (11·02–11·42) | 10·65 (10·46–10·85) | 11·10 (10·91–11·29) | 9·71 (9·54–9·88) |
| 25–29 | 11·02 (10·85–11·19) | 10·61 (10·45–10·77) | 10·67 (10·51–10·83) | 10·03 (9·87–10·19) |
| 30–34 | 10·86 (10·72–11·01) | 10·45 (10·31–10·59) | 10·24 (10·11–10·38) | 9·83 (9·70–9·97) |
| 35–39 | 11·02 (10·88–11·19) | 10·65 (10·52–10·78) | 10·44 (10·31–10·57) | 9·90 (9·77–10·03) |
| 40–44 | 11·08 (10·94–11·15) | 10·78 (10·64–10·91) | 10·62 (10·49–10·75) | 9·97 (9·84–10·10) |
| 45–49 | 10·39 (10·24–10·54) | 10·01 (9·86–10·15) | 10·04 (9·90–10·18) | 9·50 (9·37–9·64) |
| 50–54 | 9·84 (9·68–10·0) | 9·80 (9·65–9·95) | 9·65 (9·50–9·79) | 9·07 (8·93–9·21) |
| 55–59 | 9·77 (9·59–9·95) | 9·54 (9·37–9·71) | 9·45 (9·29–9·62) | 8·81 (8·66–8·96) |
| 60–64 | 9·22 (9·03–9·42) | 9·04 (8·85–9·24) | 9·28 (9·09–9·48) | 8·45 (8·27–8·64) |
| Total | 4·60 (4·58–4·62) | 4·45 (4·43–4·47) | 4·55 (4·53–4·57) | 4·23 (4·21–4·26) |

*Data are expressed in percentage. Values in parentheses represent 95% confidence intervals.

**Table S6. Estimated additional cost of fluoroquinolone use for URI in ambulatory care (in million USD)***

| **Age group (year)** | **2013** | **2014** | **2015** | **2016** |
| --- | --- | --- | --- | --- |
| 0–4 | 1·1 (1·0–1·2) | 0·8 (0·8–0·9) | 0·6 (0·5–0·6) | 0·5 (0·4–0·5) |
| 5–9 | 1·3 (1·2–1·5) | 1·2 (1·1–1·3) | 1·3 (1·2–1·5) | 1·1 (1·0–1·2) |
| 10–14 | 2·0 (1·9–2·1) | 1·9 (1·7–2·0) | 1·9 (1·8–2·1) | 1·5 (1·4–1·6) |
| 15–19 | 5·8 (5·6–6·0) | 4·2 (4·0–4·4) | 4·7 (4·6–4·9) | 4·0 (3·8–4·1) |
| 20–24 | 7·5 (7·3–7·8) | 5·1 (4·9–5·2) | 4·9 (4·7–5·0) | 4·0 (3·8–4·1) |
| 25–29 | 10·0 (9·8–10·3) | 7·4 (7·2–7·6) | 6·9 (6·7–7·1) | 5·5 (5·4–5·7) |
| 30–34 | 13·9 (13·5–14·2) | 10·8 (10·5–11·1) | 10·5 (10·3–10·8) | 8·7 (8·5–8·9) |
| 35–39 | 17·0 (16·6–17·4) | 13·7 (13·4–14·0) | 13·2 (12·9–13·5) | 10·5 (10·2–10·8) |
| 40–44 | 18·2 (17·8–18·6) | 16·3 (15·9–16·7) | 17·2 (16·8–17·6) | 13·7 (13·4–14·1) |
| 45–49 | 14·5 (14·1–14·9) | 13·2 (12·9–13·6) | 14·4 (14·0–14·8) | 13·6 (13·3–14·0) |
| 50–54 | 13·4 (13·0–13·8) | 12·4 (12·0–12·8) | 13·8 (13·4–14·2) | 11·8 (11·4–12·2) |
| 55–59 | 14·4 (13·9–14·9) | 10·8 (10·5–11·2) | 11·7 (11·3–12·1) | 10·5 (10·2–10·9) |
| 60–64 | 16·3 (15·7–17·0) | 10·3 (9·9–10·7) | 10·1 (9·7–10·5) | 8·1 (7·7–8·4) |
| Total | 135·4 (131·2–139·7) | 108·1 (104·9–111·4) | 111·2 (107·9–114·6) | 93·5 (90·6–96·5) |

*Values in parentheses represent 95% confidence intervals.
